# *AtMYB14* Regulates Cold Tolerance in *Arabidopsis*

**DOI:** 10.1007/s11105-012-0481-z

**Published:** 2012-06-22

**Authors:** Yan Chen, Zhangliang Chen, Juqing Kang, Dingming Kang, Hongya Gu, Genji Qin

**Affiliations:** 1 College of Agronomy and Biotechnology, China Agricultural University, Beijing, 100094 China; 2 The National Plant Gene Research Center (Beijing), Beijing, 100101 China; 3 State Key Laboratory for Protein and Plant Gene Research, Peking University, Beijing, 100871 China

**Keywords:** *AtMYB14*, R2R3-type MYB transcription factor, Cold tolerance, *CBF* genes, *Arabidopsis*

## Abstract

Low temperature affects plant growth and crop productivity. The *CBF* genes are a class of transcription factors that play important roles in cold response. Here we report that AtMYB14 participates in freezing tolerance in *Arabidopsis* by affecting expression of *CBF* genes. The *AtMYB14* gene was down-regulated by cold treatment. *AtMYB14* encodes a nuclear protein that functions as an R2R3-MYB transcription activator. Knock-down of *AtMYB14* by artificial microRNA increased the tolerance to freezing stress. Both the *CBF* genes and the downstream genes were induced to a much higher level in *AtMYB14* knock-down plants than in wild type under cold treatment. Our results suggest that AtMYB14 plays an important role in the plant response to cold stress.

## Introduction

Low temperature is one of the most stressful environmental factors affecting plant growth and development. Cold temperature also affects the distribution of plant species and productivity of crops (Thomashow 1998; Jiang et al. 2011; Liu et al. 2012). Plants have evolved efficient mechanisms to tolerate low temperature stress for surviving freezing periods. In the past few decades, researchers have found that many proteins are induced in the response to freezing stress. Among these, several transcriptional factors play very important roles in cold tolerance. The C-repeat binding factor (CBF) proteins, including CBF1 (DREB1b), CBF2 (DREB1c), and CBF3 (DREB1a), are pivotal transcriptional factors in this process (Stockinger et al. 1997; Liu et al. 1998). After induction by low temperature, CBF proteins regulate the expression of numerous cold responsive (*COR*) genes and increase the freezing tolerance of plants by binding to C-repeat/dehydration-responsive (CRT/DRE) elements (A/GCCGAC) in the promoter regions of these genes (Baker et al. 1994; Yamaguchi-Shinozaki and Shinozaki 1994; Thomashow 1999). The three *CBF* genes, which all belong to the AP2/EREBP gene family (Stockinger et al. 1997; Liu et al. 1998), play different roles in the cold response pathway, although they are all induced by cold stress (Novillo et al. 2004, 2007), suggesting a complex cold response signaling network. Downstream genes in the cold stress response include *KIN1* (Kurkela and Franck 1990), *COR47* (Gilmour et al. 1992), *COR15A* (Lin and Thomashow 1992), and *RD29A* (Nordin et al. 1993), which are all *COR* genes. These genes are activated by CBF transcription factors in diverse plant species (Liu et al. 2012; Wang et al. 2011; Zhang et al. 2011).

*CBF* genes are tightly regulated. The expression of *CBF3* is activated by a transcription factor called ICE1 (inducer of CBF expression 1). In contrast to *CBF3*, which is induced in cold stress, *ICE1* is expressed constitutively in *Arabidopsis* (Chinnusamy et al. 2003). ICE1 is a MYC-like transcription factor that contains a bHLH domain. ICE1 binds to the MYC recognition elements CANNTG in the promoter region of *CBF3* (Chinnusamy et al. 2003). Overexpression of *ICE1* causes elevated expression of *CBF3*, *CBF2* and downstream genes in cold stress, and thus enhances freezing tolerance to low temperature. Other proteins regulate the expression of *CBF* genes through interaction with ICE1. HOS1 (high expression of osmotically responsive gene 1)—a RING E3 ligase—interacts with ICE1 and mediates the ubiquitination and degradation of ICE1, which negatively regulates *CBF* genes (Lee et al. 2001; Dong et al. 2006). Genetic analysis shows that the transgenic plants in which HOS1 is over-expressed are hypersensitive to cold stress (Dong et al. 2006), whereas *hos1* mutants show enhanced expressions of *CBF* genes and their target genes in cold stress (Lee et al. 2001). SIZ1 (SAP and Miz1)—a SUMO (small ubiquitin-related modifier) E3 ligase—regulates ICE1 positively by adding SUMO to K393 of ICE1 (Gilmour et al. 1992). The *siz1* mutant shows reduced expressions of *CBF* genes and their target genes, and thus is sensitive to freezing. A MYB transcription factor AtMYB15 regulates *CBF* genes negatively. *AtMYB15* loss-of-function mutants increase tolerance to cold stress through elevating the expression of *CBF* genes. AtMYB15, interacting with ICE1, binds to MYB recognition sequences in the promoter regions of *CBF* genes and down-regulates *CBF* genes (Agarwal et al. 2006).

In recent years, MYB transcription factors have been found to regulate many physiological and biochemical processes, such as secondary metabolism, plant development and stress response (Kranz et al. 1998; Stracke et al. 2001; Chen et al. 2006a). MYB proteins belong to the largest family of transcription factors in *Arabidopsis* (Riechmann and Ratcliffe 2000). The DNA-binding domains of MYB proteins contain one to three imperfect repeats, named R1, R2 and R3, respectively. These repeats form a helix-turn-helix structure comprising 51–53 amino acids (Stracke et al. 2001). Among 198 *MYB* genes in *Arabidopsis*, 126 encode R2R3-MYB transcription factors, 5 encode R1R2R3-MYB proteins, 64 encode other MYB-related proteins, and 3 encode atypical MYB proteins (Chen et al. 2006a).

Many genes participate in the complex network of plant cold responses; however, the mechanisms by which plants respond to cold stress remain elusive. We previously found that many MYB genes responded to stress treatments (Chen et al. 2006a). In this study, we identified a MYB transcription factor, AtMYB14, as being involved in cold tolerance in *Arabidopsis*. Knock-down of *AtMYB14* by artificial microRNA led to increased tolerance to low temperature. The expression of *CBF* genes and cold-response downstream genes were increased to a much higher degree in *AtMYB14* knock-down plants than in wild type under cold treatment. Our results indicate that AtMYB14 is an important element in the signaling pathway of cold tolerance.

## Materials and Methods

### Plant Materials and Growth Conditions

*Arabidopsis thaliana* ecotype Columbia-0 (col-0) was used. Plants were grown as described previously (Qin et al. 2005). Briefly, seeds were plated on 1/2 × MS medium (half strength Murashige and Skoog medium) containing 1 % sucrose. The plates were kept at 4 °C in the dark for 3 days for synchronization. The plates were transferred to a chamber at 22 °C ± 2 °C under long-day conditions (16 h light / 8 h dark). After 7 days the seedlings were transferred to soil and placed in a greenhouse at 22 °C ± 2 °C under long-day conditions (16 h light / 8 h dark).

### Cold Treatment and Freezing Tolerance Test

For cold treatment in experiments to determine *AtMYB14* regulation by cold stress, 2-week-old wild-type plants on 1/2 × MS medium were placed in a chamber at 0 °C under continuous light for 0, 0.5, 1, 3, 6, 12, or 24 h.

The freezing tolerance test of Arabidopsis was described previously with some modifications (Zhu et al. 2004). Three-week-old plants in soil in the greenhouse were placed in a freezing chamber at −1 °C. The chamber cooled at a rate of −1 °C/h, then held at −8 °C. After exposure to −8 °C for 2 h, plants were transferred to a greenhouse at 22 °C under long-day conditions. The test was conducted in the dark in a freezing chamber. Survival rates of plants were assessed after 7 days.

### RNA extraction, Reverse Transcription and Real-Time Quantitative PCR

Total RNA was extracted from 2-week-old Arabidopsis seedlings using Trizol reagent. The total RNA was treated with DNase I (TaKaRa, Tokyo, Japan) and then reverse-transcribed according to the producer’s manual and as described previously (Liu et al. 2008). The real-time quantitative PCR conditions were as described by Xing et al. (2007). Briefly, the reaction was performed on an Option 2 Continuous Fluorescence Detector (MJ Research, Waltham, MA) and a SYBR Green realtime PCR Master Mix (TOYOBO, Tokyo, Japan) was used. The real-time PCR products were assessed by melting curve and gel electrophoresis to ensure the specificity of the reaction. Three technical replicates were carried out for each biological sample. Cycling conditions were 95 °C for 2 min, 95 °C for 20 s, 55 °C for 20 s, and 72 °C for 20 s. The relative expression level of each gene was calculated using the 2^-ΔΔCT^ method. The Arabidopsis elongation factor gene *EF1-1* was used as an internal control. The gene-specific primers for *EF1-1* (*AT1G07930*) were (5′-CCT CCC AGG CTG ATT GTG CT-3′ and 5′-TAT TTG GGG GTG GTG GCA TC-3′). The other gene-specific primers are as follows: *AtMYB14* (5′-GTT AAC GGA ATT AAC GAG ACC ACA A-3′ and 5′-AAAC TAT CAT CTA TCA AGG CAG AAA-3′), *CBF1* (5′-CTG AAG TGA GAG AGC CAA AC-3′ and 5′-AGT CAG CGA AGT TGA GAC AT-3′), *CBF2* (5′-CTA TTT ATA CGC CGG AAC AG-3′ and 5′-GCC ATG TTA TCC AAC AAA CT-3′),* CBF3* (5′-TTT CAG GAT GAG ATG TGT GA-3′ and 5′-CTT CTG CCA TAT TAG CCA AC-3′)*, RD29A* (5′-ATC ACT TGG CTC CAC TGT TGT TC-3′ and 5′-ACA AAA CAC ACA TAA ACA TCC AAA GT-3′), *KIN1* (5′-ACC AAC AAG AAT GCC TTC CA-3′ and 5′-CCG CAT CCG ATA CAC TCT TT-3′), *COR15A* (5′-GGC CAC AAA GAA AGC TTC AG-3′ and 5′-CTT GTT TGC GGC TTC TTT TC-3′), *COR47* (5′-AAG CTT CAC CGA TCC AAC AG-3′ and 5′-TAC CGG GAT GGT AGT GGA AA-3′).

### Subcellular Localization

For subcellular localization, the full-length cDNA of *AtMYB14* was amplified using the primers *AtMYB14*-F (5′-GAA GAT CTA TGG GAA GAG CAC C-3′) and *AtMYB14*-R (5′-GCT CTA GAT TAA AAC TCG GGT A-3′). The PCR products were digested with *Bgl* II and *Xba* I, and then cloned into the vector pRTL-GFP (Yi et al. 2002). The construct was bombarded into epidermal cells of onions as described previously (Li et al. 2006a).

### Transactivation Activity Assays

The transcription activity of AtMYB14 was examined by a yeast one-hybrid assay using the yeast strain EGY48. We evaluated AtMYB14 activity using deletion mutants: the N-terminal region of 1–115 amino acids containing the MYB domain, a short (25 aa) region corresponding to amino acids 224–249 containing the conserved M(E/D)FWFD motif in the C-terminal region, the C-terminal region of amino acids 116–223 without the short 25 aa region, and the C-terminal region of amino acids 116–249. The deletions of AtMYB14 were amplified using the following primers: *AtMYB14*-1/115 (5′-GGA ATT CAT GGG AAG AGC AC-3′ and 5′-CCG CTC GAG TCT TTT CTT CA-3′), *AtMYB14*-224/249 (5′-CCG GAA TTC ATG AAT GAT GAC ATG GA-3′, *AtMYB14*-116/223 (5′-GGA ATT CAT GCT CAG CAA AA-3′ and 5′-CCG CTC GAG ATA CAA CTT AG-3′), and 5′-CCG CTC GAG TTA AAA CTC GG-3′), *AtMYB14*-116/249 (5′-GGA ATT CAT GCT CAG CAA AAA TCT AAA C-3′ and 5′-CCG CCG CTC GAG TTA AAA CTC GGG TAT G-3′). The products were digested with *Eco*R I and *Xho* I and cloned into the vector of pYF503 (Ye et al. 2004). Transactivation activity assays were performed as described previously (Li et al. 2006b).

### Construction of *Pro*_*AtMYB14*_::GUS and Histochemical GUS Assays

For detection of AtMYB14 promoter activity, the promoter was amplified and cloned in front of a β-glucuronidase (GUS) reporter gene as follows. The primers *AtMYB14*-pro-F (5′-GGA TCC TGA TGG AAT TAG ATA TCG ACG-3′) and *AtMYB14*-pro-R (5′-ACT AGT GAG AGC TCG CCA ATT AGA ATG-3′), were used for amplification. The PCR products were digested with *Bam*H I and *Spe* I, and then cloned into the vector pCAMBIA1381Xa to generate *Pro*_*AtMYB14*_::GUS. GUS activity was analyzed as described previously (Chen et al. 2006b) using 2-, 4-, 8-, and 16-day-old seedlings. Briefly, whole seedlings were infiltrated in GUS staining solutions containing 0.5 mg/ml 5-bromo-4-chloro-3-indolyl glucuronide in 0.1 M Na_2_HPO_4_, pH 7.0, 10 mM Na_2_EDTA, 0.5 mM potassium ferricyanide/ferrocyanide, and 0.06 % Triton X-100 and incubated at 37 °C overnight. The stained plants were then cleared in 70 % ethanol.

### *AtMYB14* Overexpression and Artificial microRNA of *AtMYB14*

To generate the *AtMYB14* overexpression construct, the full-length cDNA of *AtMYB14* was amplified using primers *AtMYB14*-OX-F (5′-CTC GAG AAA AAG AAT GGG AAG AGC ACC-3′) and *AtMYB14*-OX-R (5′-TCT AGA AAA TCA AAA TTA AAA CTC GGG-3′). The amplified fragments were digested by *Xho* I and *Xba* I , and then cloned into the vector pJim19, in which *AtMYB14* is driven by the CaMV35S promoter. Transformation mediated by *Agrobacterium tumefaciens* strain GV3101 into Arabidopsis plants was carried out by the floral dipping method as described previously (Qin et al. 2005).

The engineering of artificial microRNA was described by Schwab et al. (2006). For construction of artificial microRNA *AtMYB14*, the Web MicroRNA Designer (http://www.weigelworld.org) was used to identify the sequences. The primers used were as follows: PBS300-A (5′-CTG CAA GGC GAT TAA GTT GGG TAA C-3′), PBS300-B (5′-GCG GAT AAC AAT TTC ACA CAG GAA ACA G-3′), I miR-s (5′-gaT AA TTA TCT ACC GAT ATG GCG tct ctc tttt gta ttc c-3′), II miR-a (5′-gaC GCC ATA TCG GTA GAT AAT Tat caa aga gaa tca atg a-3′), III miR-s (5′-gaC ACC ATA TCG GTT GAT AAT Tat cac agg tcg tga tat g-3′), IV miR-a (5′-gaT AAT TAT CAA CCG ATA TGG TGt cta cat ata tat tcc t-3′). The amiRNA precursor was cloned into pBS and confirmed by sequencing. The pBS-amiRNA14 was digested with *Spe* I and *Xho* I, and the target DNA fragment was cloned into pJim19 to generate the construct pJim19-amiRNA14, which was then transformed into Arabidopsis plants by floral dipping (Qin et al. 2005).

T2 seeds from *AtMYB14* overexpression lines and amiRNA14 T1 transgenic lines were plated on 1/2MS medium containing 50 μg/ml kanamycin. The numbers of green and yellow seedlings were counted. If the ratio of green to yellow seedlings from T1 progenies was 3:1, the T1 line was selected as a transgenic line with a single copy of the T-DNA. The green plants were then transferred to soil. The T3 seeds were again plated on 1/2MS medium containing 50 μg/ml kanamycin. Lines with no segregation were selected as *AtMYB14* overexpression or amiRNA14 homozygous lines.

## Results

### Expression of *AtMYB14* is Down-Regulated by Cold Stress

**Fig. 1:**
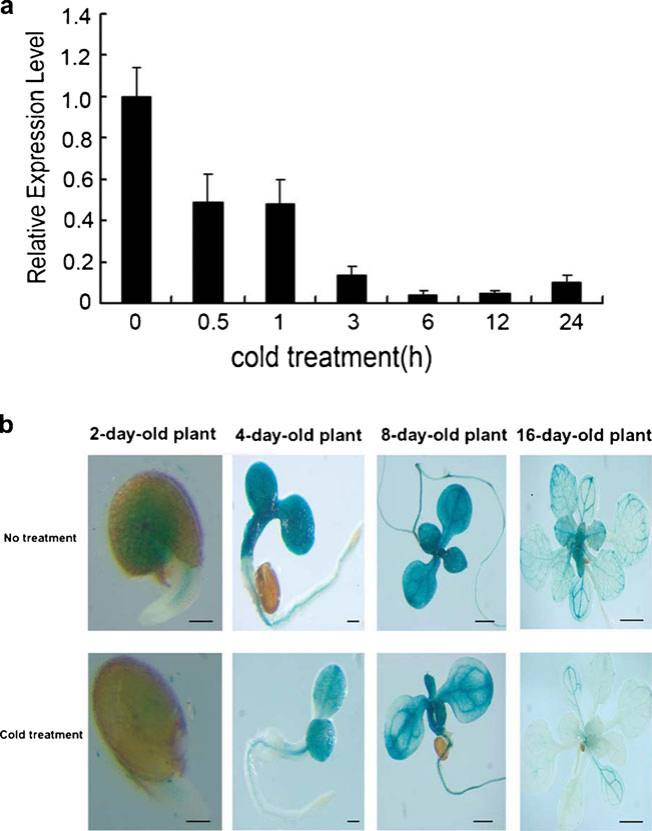
Expression of *AtMYB14* is down-regulated by cold treatment. **a** *AtMYB14* transcript levels of 2-week-old wild type plants under 0 °C treatment were revealed by real-time quantitative PCR analysis. *EF1-1* was used as an internal control.* Error bars* SD of biological replicates (*n* = 3). **b** GUS staining of *Pro*_*AtMYB14*_::GUS transgenic plants with or without 0 °C treatment for 24 h. The transgenic plants were treated at 4 °C or 22 °C at the end of 2, 4, 8 and 16 days after germination.* Bars* 100 μm in 2- and 4-day-old plants, 400 μm in 8-day-old plants, and 800 μm in 16-day-old plants

We have previously identified 163 *MYB* genes and found that many of them are regulated by stress treatments (Chen et al. 2006a). In order to search for possible functions of MYB transcription factors in cold tolerance, we first examined changes in their expression under cold treatment using real-time quantitative PCR. The results showed that one transcription factor, *AtMYB14*, was significantly down-regulated by cold stress, whereas the relative expression level of *AtMYB14* at normal temperature (22 °C) at different times showed no changes in expression (data not shown), indicating that changes in expression of *AtMYB14* were not due to circadian rhythm. As shown in Fig. 1, *AtMYB14* transcripts decreased rapidly during 0 °C treatment. The level of gene expression was reduced by more than half after cold treatment for 30 min. After 6 h at 0 °C, expression of *AtMYB14* reached its lowest level (Fig. 1a). To further confirm the down-regulation of *AtMYB14* by cold stress, we cloned the promoter region of *AtMYB14* and fused it to the *uidA* gene to generate the *Pro*_*AtMYB14*_::GUS construct. *Pro*_*AtMYB14*_::GUS transgenic plants were treated at 0 °C for 24 h; GUS staining showed that the GUS activity decreased significantly under cold treatment in the seedling stage (Fig. 1b). Taken together, these results indicate that *AtMYB14* is down-regulated by low temperature.

### *AtMYB14* Encodes an R2R3-MYB Domain Protein

**Fig. 2:**
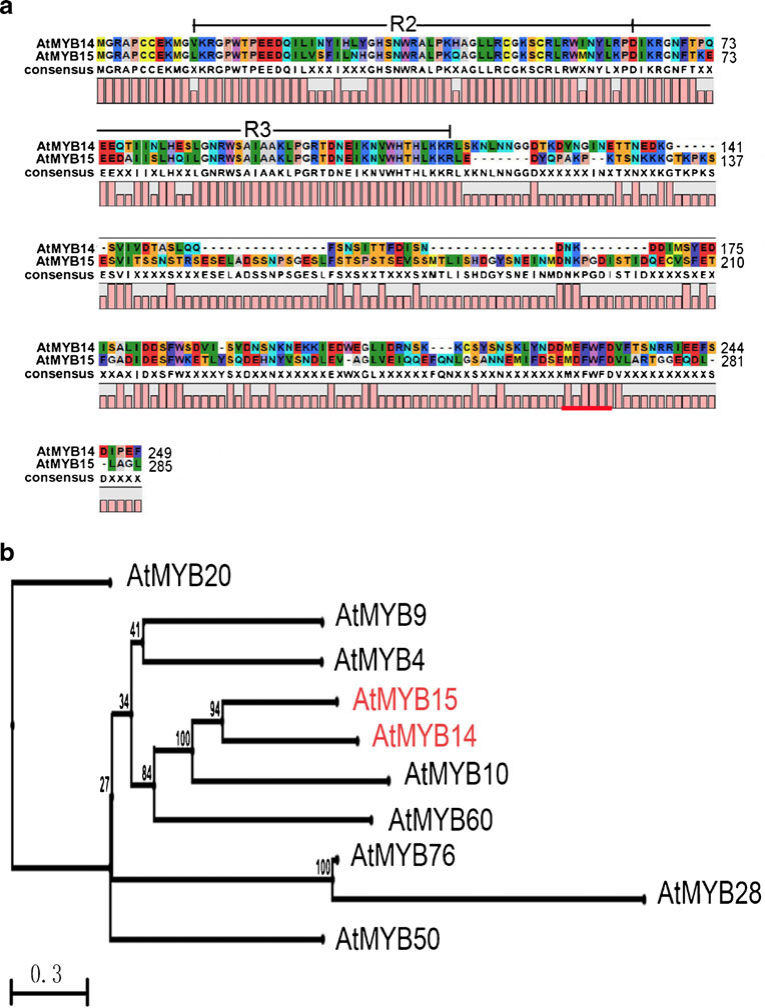
Sequence analysis of AtMYB14. **a** Amino acid alignment of AtMYB14 and AtMYB15.* Black lines* R2 and R3 MYB domains. Identical amino acid residues are shown in the same* color*. The conserved motif M(E/D)FWFD in the C-terminal is underlined by a* thick red line*. **b** Phylogenetic tree of AtMYB14 and some other R2R3-MYB proteins from *Arabidopsis*. The phylogenetic tree was constructed on the basis of N-terminal domains of R2R3-MYB using the CLC sequence viewer program (http://www.clcbio.com). AtMYB14 and AtMYB15 are grouped together

Bioinformatics analysis revealed that *AtMYB14* encodes a protein of 249 amino acids that contains an R2R3-MYB domain (Fig. 2a). The MYB domain is a DNA binding domain that consists of one to three imperfect repeats named R1, R2 and R3 (Chen et al. 2006a). Within the large MYB protein family in *Arabidopsis*, AtMYB14 protein sequence shares the highest similarity with AtMYB15 in the MYB repeats. Their MYB domains share 82.5 % amino acid sequence identity (Fig. 2a). Interestingly, AtMYB14 contains one C-terminal motif M (E/D) FWFD similar to that of AtMYB15 involved in cold tolerance (Fig. 2a) (Agarwal et al. 2006). According to sequence similarity, AtMYB14 is grouped with AtMYB15 in the phylogenetic tree (Fig. 2b).

### AtMYB14 is a Nuclear Protein and has Transcriptional Activation Activity

**Fig. 3:**
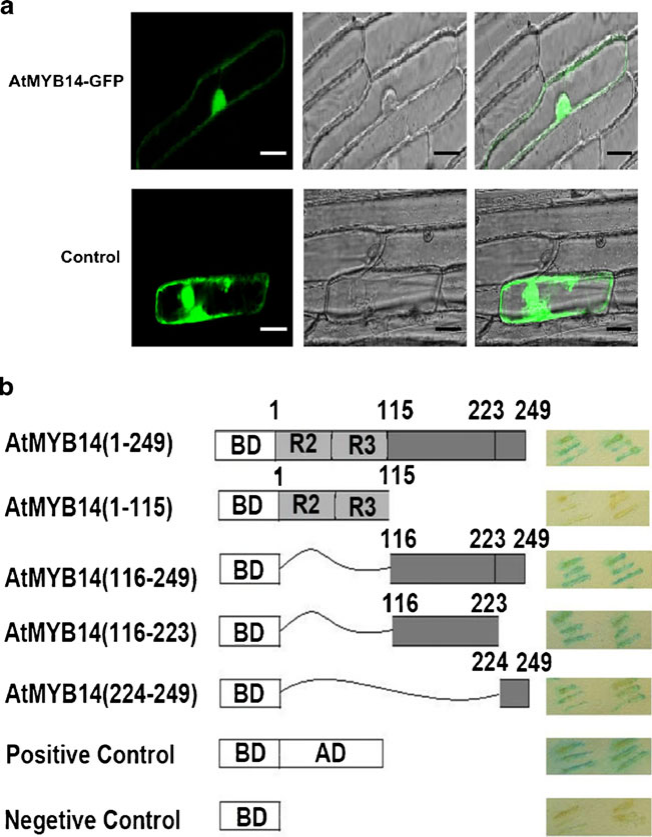
Subcellular localization and transactivation activity of AtMYB14. **a** Subcellular localization of AtMYB14. Images taken using a fluorescence microscope (*left*), bright microscope (*middle*) were merged (*right*).* Bar* 50 μm. **b** Transactivation of AtMYB14 in yeast. Full-length and deletion mutants of *AtMYB14* cDNA are illustrated on the* left* and transactivation activity on the* right*. Empty pYF503 was used as the negative control and pYF504 used as the positive control

In order to test the subcellular localization of the AtMYB14 protein, an AtMYB14-GFP construct was generated by fusing full-length *AtMYB14* cDNA in frame with the N-terminus of the green fluorescent protein (*GFP*) gene. The fusion protein was driven by a CaMV 35S promoter in the AtMYB14-GFP construct. This construct was bombarded into onion epidermal cells. The control construct in which the *GFP* gene was driven by the CaMV 35S promoter was also transformed by bombardment. In AtMYB14-GFP transgenic cells, strong GFP fluorescence was found in the nucleus, and a weak fluorescence outside the nucleus was also observed (Fig. 3a), whereas the GFP control was localized throughout the cell (Fig. 3a). These results indicate that AtMYB14 is a nuclear protein.

To examine the transcription activity of AtMYB14, we used a yeast one-hybrid system (Ye et al. 2004), in which full-length AtMYB14 and a series of deletions were fused to the GAL4 DNA-binding domain and then co-transformed with the *LacZ* reporter into yeast cells. The results showed that the full-length protein displayed a high transactivation activity (Fig. 3b). This suggests that AtMYB14 might be a transcriptional activator. No GUS activity could be detected for the N-terminal region of 1–115 amino acids containing the MYB domain (similar activity to the negative control; Fig. 3b). The C-terminal region of 116–249 amino acids showed high GUS activity, suggesting that this region may contain the activation domain. Interestingly, the short region comprising amino acids 224–249 containing the conserved C-terminal M(E/D)FWFD motif showed GUS activity. The C-terminal region of amino acids 116–223 without this short region also had transactivation activity (Fig. 3b), which differs from that observed in AtMYB15 (Chen et al. 2006b). The results suggest that AtMYB14 may be a transcriptional activator and that amino acids 116–249 in the C-terminal region are responsible for the transactivation activity.

### Expression Pattern of *AtMYB14*

**Fig. 4:**
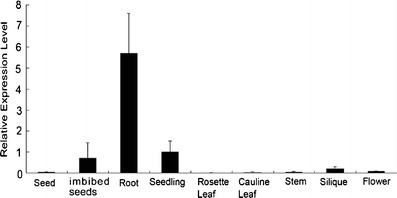
Expression pattern of *AtMYB14*. Analysis of relative *AtMYB14* transcript levels in different tissues by real-time quantitative PCR. *EF1-1* was used as an internal control. The expression level of *AtMYB14* in seedlings was set to 1.0.* Error bars* SD of biological replicates (*n* = 3)

To elucidate the expression pattern of *AtMYB14*, we first analyzed its transcript level in different tissues using real-time quantitative PCR in *Arabidopsis*. *AtMYB14* was expressed in imbibed seeds, 5-day-old roots, the shoots of 2-week-old seedlings, siliques and flowers, with the highest level in the roots (Fig. 4). Strong GUS activity was detected in hypocotyls, cotyledons and roots in 4-day-old *Pro*_*AtMYB14*_::GUS transgenic seedlings and 8-day-old *Pro*_*AtMYB14*_::GUS transgenic seedlings (Fig. 1b), which was consistent with the real-time PCR results. GUS staining in 16-day-old seedlings was faded in the roots and old leaves when compared with that observed in 8-day-old seedlings, indicating that *AtMYB14* may be developmentally regulated.

### Knock-down of *AtMYB14* Increases Tolerance to Cold Stress

**Fig. 5:**
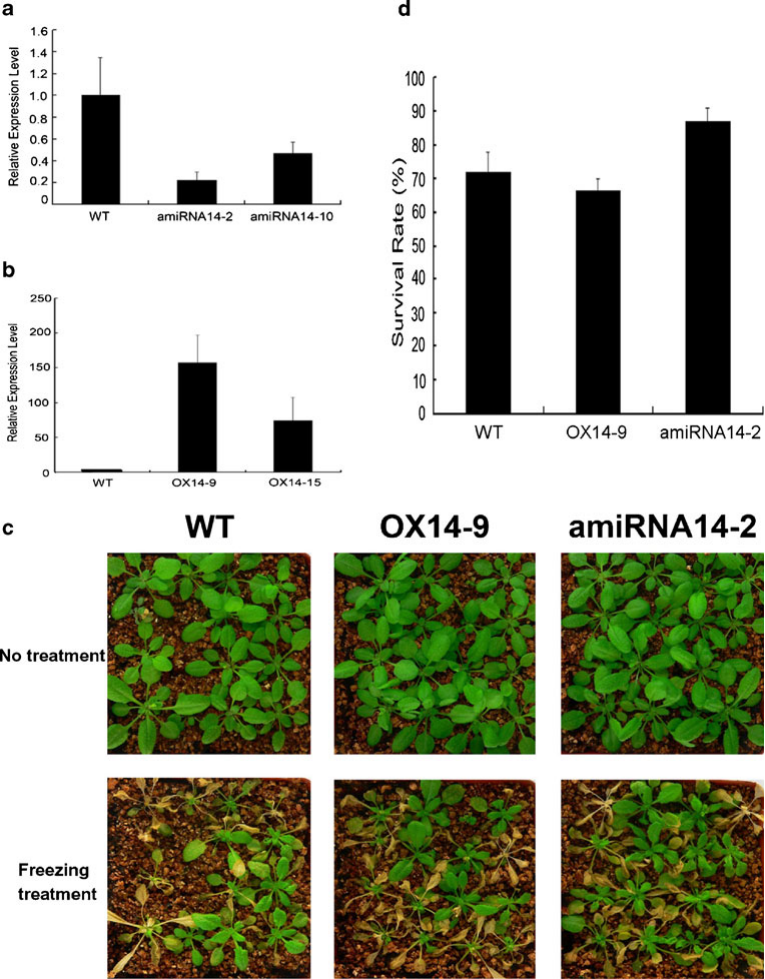
Increased freezing tolerance of the *AtMYB14* knock-down lines. **a** Real-time quantitative PCR analysis of relative *AtMYB14* transcript levels in amiRNA14 lines; the expression level of *AtMYB14* in wild type plants was set to 1.0. **b** Real-time quantitative PCR analysis of relative *AtMYB14* transcript levels in OX14 lines; the expression level of *AtMYB14* in wild type plants was set to 1.0. **c** Freezing test carried out using wild type, OX14-9 and amiRNA14-2 plants. All plants were grown in soil at 22 °C for 3 weeks and then transferred into a freezing chamber at −1 °C. The chamber cooled at the rate of −1 °C/h, then held at −8 °C. After exposed to −8 °C for 2 h, plants were transferred to a greenhouse at 22 °C. After 7 days, the plants were photographed. **d** Survival rates of amiRNA14-2 and OX14-9 were analyzed on the 7th day after the freezing test.* Error bars* SD of biological replicates (*n* = 3)

To investigate the function of *AtMYB14* in cold stress, we knocked down the transcript level of *AtMYB14* in transgenic plants using artificial miRNA technique. Six transgenic plants were obtained and the transcript levels of *AtMYB14* were analyzed using real-time quantitative PCR. Two independent lines—amiRNA14-2 and amiRNA14-10—showed a significant reduction in *AtMYB14* transcript levels (Fig. 5a). The expression level of *AtMYB15*, which has high similarity with *AtMYB14*, was not affected by the artificial microRNA in the amiRNA14-2 line (data not shown). T3 homozygous lines of amiRNA14-2 were selected for tolerance analysis in cold stress because of their lower *AtMYB14* transcript levels (Fig. 5a). We also generated a construct in which *AtMYB14* was driven by the CaMV 35S promoter. We obtained 11 transgenic plants and transcript levels of *AtMYB14* were analyzed. Two homozygous transgenic plants with increased *AtMYB14* expression were selected and named as OX14-9 and OX14-15 (Fig. 5b). The T3 homozygous lines of OX14-9 was chosen for cold tolerance analysis because of its higher *AtMYB1* expression level. As shown in Fig. 5c,d, following treatment of these plants at −8 °C for 2 h, the survival rate of amiRNA14-2 was 87 %, whereas the survival rate of the wild type control was only 71 %. The survival rate of OX14-9 lines was 67 %, which was not significantly different from that of the wild type control. This indicated that the amiRNA14-2 plants showed enhanced tolerance, but OX14-9 plants had no obvious difference from wild-type plants in cold tolerance. These results indicate that *AtMYB14* may be involved in signal transduction in the plant cold response.

### Knockdown of *AtMYB14* Increases Expression of *CBF* Genes Under Cold Treatment

**Fig. 6:**
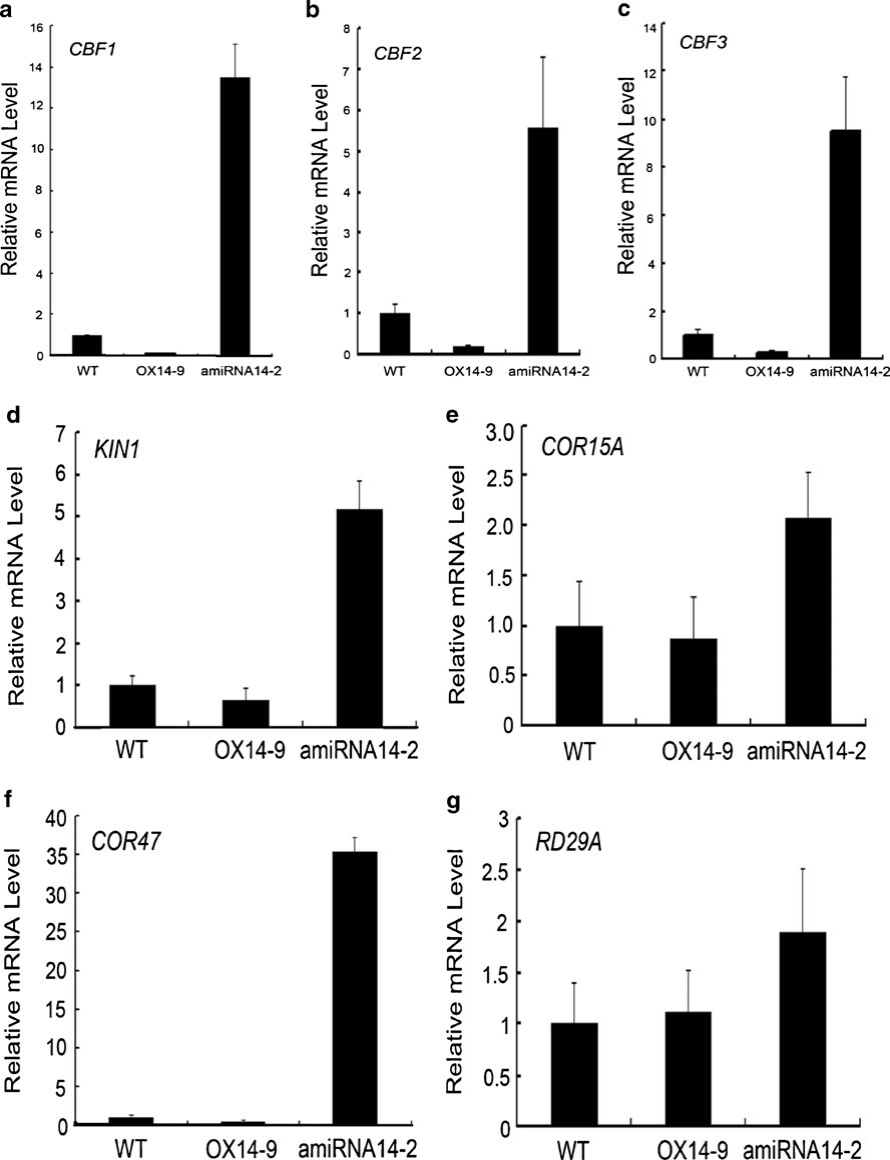
Relative mRNA levels of cold responsive genes examined by real-time quantitative PCR. Expression levels of *CBF1* (**a**), *CBF2* (**b**), and *CBF3* (**c**) were increased to a much greater extent in amiRNA14-2 than in wild-type or OX14-9 lines under cold treatment. The expression levels of the downstream genes such as *KIN1* (**d**), *COR15A* (**e**)*, COR47* (**f**), and *RD29A* (**g**) in amiRNA14-2 lines were also elevated to a much higher level than those in wild-type or OX14-9 lines under low temperature. The gene expression level in wild type plants was set to 1.0.* Error bars* SD of biological replicates (*n* = 3)

To determine the molecular mechanisms by which AtMYB14 regulates cold tolerance, we examined changes in expression of *CBF* genes, which are known regulators of cold response. When amiRNA14-2, OX14-9 and wild-type plants were treated at 0 °C for 24 h, the expression levels of all three *CBF* (*CBF1*,* CBF2*, and *CBF3*) were increased much more in amiRNA14-2 than in wild-type or OX14-9 lines (Fig. 6a–c). The expression levels of these three genes in OX14-9 lines were a little lower than those in wild-type plants (Fig. 6a–c). These results suggest that *AtMYB14* may affect upstream genes in the cold signaling pathway. We next analyzed changes in the expression of *CBF* downstream genes. Consistent with the changes in *CBF* genes, the expression levels of *CBF* downstream genes, including *KIN1*,* COR15A*,* COR47*, and *RD29A* in amiRNA14-2 lines were elevated to levels higher than those in wild-type or OX14-9 lines when they were treated at 0 °C for 24 h (Fig. 6d–g). These results suggest that the increased freezing tolerance in amiRNA14-2 plants resulted from the much higher expression of *CBF* genes and their downstream genes under cold treatment.

## Discussion

Plants survive periods of low temperature in their life cycles by changing gene expression profiles to make physiological adjustments. Some important transcription factors and many *COR* genes are induced in cold stress to realize these physiological alterations (Thomashow 1999; Viswanathan and Zhu 2002l; Xiong et al. 2002). *CBF* genes are induced by exposure to low temperature and encode pivotal transcription factors that activate the downstream *COR* genes in the cold signaling pathway (Stockinger et al. 1997; Gilmour et al. 1998). In our study, we found that AtMYB14, an R2R3-type transcription factor with transactivation activity, participated in cold tolerance by affecting *CBF* genes. The expression of *AtMYB14* was down-regulated by cold treatment. Knock-down of *AtMYB14* by artificial microRNA in transgenic plants caused higher resistance to cold stress through regulating *CBF* genes. We suggest that AtMYB14 plays an important role in cold response in Arabidopsis.

AtMYB14 was found to have a high similarity with AtMYB15 in MYB domains. AtMYB15 was found to bind to the promoters of *CBF* genes and functioned as a negative regulator of their expression (Agarwal et al. 2006). Although both MYB proteins played a negative role in cold response, there were differences. First, *AtMYB15* is up-regulated in cold stress (Agarwal et al. 2006), whereas *AtMYB14* is down-regulated by cold treatment. It is logical that plants down-regulate negative regulators when facing cold stress and thus release expression of genes that protect against cold stress (Lee et al. 2001). It is still unknown why plants elevate the expression of *AtMYB15* in low temperature since it is a negative regulator. In this study, we found that expression of *AtMYB15* was not altered in either *AtMYB14* overexpression lines or knock-down lines. This indicated that *AtMYB15* expression is not affected by changes in expression of *AtMYB14*. Second, the downstream genes *RD29A* and *COR15A* showed no changes in *AtMYB15* overexpression and knock-down lines (Lin and Thomashow 1992; Lee et al. 2001; Agarwal et al. 2006), whereas in *AtMYB14* knock-down lines these genes are highly induced as expected. These differences suggest that the functional mechanisms of AtMYB14 and AtMYB15 in cold tolerance differ. The C-terminal 116–223 amino acids in AtMYB14 had low similarity to the corresponding region of AtMYB15. This region showed high transactivation activity (Fig. 3b). This may explain the molecular basis of the difference between AtMYB14 and AtMYB15.

The results of transactivation activity assays in yeast reveal that AtMYB14 may be a transcriptional activator, but the knock-down lines show increased tolerance to freezing by inducing expression of *CBF* genes and their downstream genes in cold stress. This sounds conflicting, but is not unusual in plants. For example, WRKY48 has been reported to have a powerful transcriptional activity, but is a negative regulator in plant basal defense (Xing et al. 2008). A possible reason for an activator functioning as a repressor is that AtMYB14 may not regulate *CBF* genes directly, but might activate other transcription factors that repress the expression of *CBF* genes. Alternatively, AtMYB14 may interact with some repressor and recruit it to the promoter regions of target genes. Although the actual mechanisms by which AtMYB14 regulates cold tolerance by down-regulating *CBF* genes are still unknown, the data reported here suggest strongly that it is involved in the plant response to low temperature.

In summary, AtMYB14 is transcription activator that plays an important role in cold tolerance by affecting *CBF* genes. This provides new information to increase our understanding of the complex network of transcriptional control in the plant response to cold stress.
